# Addressing the challenges and constraints of social protection policies for Peruvian women domestic workers: the ANITA project study protocol

**DOI:** 10.1136/bmjopen-2024-088921

**Published:** 2025-03-06

**Authors:** Janeth Tenorio-Mucha, Sebastián García, Jill Portocarrero, Maria Lazo-Porras, Karina Romero Rivero, David Vera Tudela, María Kathia Cárdenas, Silvana Pérez-León, Nathaly Aya Pastrana, Maria Sofia Cuba-Fuentes, Ayu Pinky Hapsari, Christopher Meaney, Andrew David Pinto, Viviana Cruzado, Archna Gupta

**Affiliations:** 1 CRONICAS Center of Excellence in Chronic Diseases, Universidad Peruana Cayetano Heredia, Lima, Peru; 2 Universidad Peruana Cayetano Heredia, Lima, Peru; 3 IMEK Centro de Investigación en Mercadeo & Desarrollo, Santiago de Cali, Valle del Cauca, Colombia; 4 Center for Research in Primary Health Care, Universidad Peruana Cayetano Heredia, Lima, Peru; 5 Upstream Lab, MAP Centre for Urban Health Solutions, Li Ka Shing Knowledge Institute, St Michael’s Hospital, Toronto, Ontario, Canada; 6 Department of Family and Community Medicine, University of Toronto, Toronto, Ontario, Canada; 7 St Michael's Hospital Department of Family and Community Medicine and St Michael’s Academic Family Health Team, Toronto, Ontario, Canada; 8 Pontificia Universidad Católica del Perú, Lima, Peru; 9 Estado Peruano Ministerio de Desarrollo e Inclusión Social, Lima, LIma, Peru

**Keywords:** Caregivers, Community-Based Participatory Research, Decision Making, Health Equity, Latin America, Research Design

## Abstract

**Abstract:**

**Introduction:**

Domestic workers (DWs) are vulnerable to precarious or informal working conditions with limited access to social protection policies such as employer-paid health insurance or retirement pensions. This study aims to examine the working conditions, health status and access to healthcare for women DWs in Peru and propose recommendations to improve their access to social protection policies.

**Methods and analysis:**

The project uses a participatory action research approach by engaging three committees: a DW co-researcher committee, an advisory committee and a steering committee. The first two include former or current DWs, while the third includes policymakers and academics. We use a sequential mixed-methods design organised in four phases: (1) secondary data analysis (n=4216): using two Peruvian national surveys to characterise working conditions, health status and access to healthcare; (2) face-to-face survey (n=448): with DWs in three cities, using respondent-driven sampling to further characterise working and health conditions and to identify factors that influence knowledge of and access to social protection policies; (3) qualitative interviews (n=30–46): with DWs, leaders of DW organisations, employers and policymakers to gather different perspectives on the facilitators and barriers to access to social protection policies; and (4) deliberative dialogues (n=14–26): with DW, leaders of DW organisations, employers, policymakers and academics to identify key barriers to the implementation of social protection policies and to develop recommendations for overcoming these barriers.

**Ethics and dissemination:**

Phase 1 and Phase 2 received ethical clearance from Universidad Peruana Cayetano Heredia in Peru and Unity Health Toronto in Canada. Phase 3 and Phase 4 received ethical clearance from PRISMA Charitable Association in Peru and Unity Health Toronto in Canada. To mobilise knowledge, in collaboration with the committees, we will co-generate policy briefs and audiovisual materials to disseminate the results from this project to different audiences and sectors.

**STRENGTHS AND LIMITATIONS OF THIS STUDY**

- The findings of the quantitative surveys and qualitative interviews will guide the identification of challenges and needs to support the development of evidence-based policy recommendations and deliberative dialogues.
- We will adopt a participatory-action research approach, working closely with domestic workers and their union leaders to conduct research that is responsive to their needs and promotes ownership of the findings and recommendations.
- We will use a respondent-driven sampling technique in three different cities in Peru to survey a diverse range of domestic workers and to capture different work characteristics and social protection needs.
- While we seek a comprehensive understanding of working and health conditions, the findings may not be generalisable to the broader population of paid domestic workers, especially given the diversity of working conditions and social protection needs.
- We will implement strict confidentiality measures, provide emotional and safety support and ensure well-trained staff promote the fair treatment of participants and reduce potential underreporting of sensitive topics.

## Introduction

 Domestic work plays a vital role in society by providing essential services such as cleaning, cooking, caring for children, the elderly and persons with disabilities or tending the gardens or pets.^1^ While unpaid domestic work is essential to household functioning, paid domestic work is critical in sustaining economies by enabling other workers to participate in the labour force. Paid domestic work also offers vital income for millions of families, yet it remains undervalued and often lacks adequate social protections.^2^

Almost 80% of paid domestic workers (DWs) worldwide are women, a result of traditional gender roles that link caregiving and household tasks to women, driving low wages and limited labour protections as the work is viewed as an extension of unpaid care.^1^ Women DWs have poor access to employer-paid health insurance, retirement pensions or unemployment compensation, which constitute social protection benefits.^3^ In the Latin American region, the provision of social protection to paid DWs is less than 50% in all countries, but there are significant variations between countries.^4 5^ The countries with higher provision of social protection through social security (between 41% and 43%) are Uruguay, Chile and Ecuador, while far behind are Bolivia (4.4%), El Salvador (1.9%) and Mexico (0.1%).^5^

In the ranking mentioned above, Peru is among the five countries with the worst access to social protection for DWs. This is despite Peru’s government endorsing the International Labour Organisation (ILO) Convention 189^6^ in 2018, which triggered a movement in the republic’s own legislature. In Peru, domestic work is now regulated by the ‘Domestic Workers Law’ (Law number 31047)^7^ approved in October 2020, which establishes that (1) a workday and week consist of 8 hours and 48 hours, respectively; (2) payment of at least the minimum wage (around US$240); (3) mandatory healthcare insurance coverage and maternity leave and (4) for DWs living in the workplace, provision of lodging and food by the employer in addition to the salary. However, 86.7% of DWs work under informal conditions and, therefore, do not benefit from the Domestic Worker Law given the absence of law enforcement.^8^

Domestic work is also a job where women are more vulnerable to exploitation, violence and segregation, which in Peru has historical roots in the slavery of the pre-republican era.^9^ Some characteristics of women DWs in Peru have been described in thesis,^10^ discussion papers^11^ and scientific articles.^12 13^ These papers highlighted the economic disadvantages and precarious working conditions women DWs experience. DWs have irregular working hours, salaries below the minimum wage, sexual harassment and various forms of discrimination (by race or ethnicity, education, social status and gender).^14^ The COVID-19 pandemic further exacerbated the challenges for DWs by adding more direct and indirect barriers to decent work and healthcare access. It is estimated that 72.1% of DWs lost their jobs between the last quarter of 2019 and the second quarter of 2020,^8^ while those who maintained their jobs experienced reduced working hours and wages or were confined at the workplace for long periods where they were prohibited from seeing family or friends.^15^

While Peru’s regulations for DWs have improved, barriers to policy implementation and enforcement remain underexplored. The constraints and challenges of social protection policies for DWs should be addressed, as they are often among the most vulnerable and marginalised workers. Moreover, their diverse experiences, shaped by factors like race or ethnicity, language and migration status, are rarely studied. Understanding these lived experiences can lead to more responsive and transformative policies.

The ANITA project, ‘Addressing the Challenges of Social Protection Policies for Peruvian Women Domestic Workers’, aims to understand their working and health conditions, identify barriers and facilitators to policy implementation and propose recommendations for improving social protection. These recommendations will consider the contexts and specific needs of DWs in three different settings in the country while concurrently considering intersecting variables (such as age, education and self-identified ethnicity or race) that influence how DWs experience their work and life. The recommendations will potentially be a reference for other low- and middle-income countries.

The ANITA project opens up a new social and research context for Peruvian DWs. We consider the project innovative because: (1) it integrates mixed methods for a comprehensive understanding of the facilitators, constraints and challenges; (2) we use a participatory action approach integrating DWs in planning, developing, analysing and disseminating findings; (3) we use an innovative recruitment technique that allows us to reach hard-to-reach populations and (4) we promote knowledge translation and a space for discussion with different stakeholders.

The ANITA project is one of the 23 projects funded by the Women RISE initiative. The Women RISE initiative was launched in March 2022 by Canada’s International Development Research Centre in partnership with the Canadian Institutes of Health Research and the Social Science and Humanities Research Council. This initiative supported action-oriented and gender-transformative research on how women’s health and work intersect and interact.^16^ We believe our project will provide new and unique evidence and propose feasible recommendations for a more inclusive and sustainable COVID-19 pandemic recovery for DWs in Peru.

## Aim and objectives

The project aims to examine the working conditions, health status and access to healthcare for women DWs in Peru and propose recommendations to improve their access to social protection policies. Our three specific research objectives are:

1. To explore and compare the working conditions, health status and access to healthcare of women DWs before, during and after the COVID-19 pandemic.
2. To identify the factors that influence women DWs’ knowledge of and access to social protection policies.
3. To co-design feasible and context-adapted recommendations to improve access to social protection policies for women DWs.

This paper aims to outline the methods that will be used to achieve these three objectives.

## Participatory action research (PAR) approach

This project uses a PAR approach, which engages participants in all aspects of the research, from establishing the research question to designing research methods, data analysis and knowledge dissemination.^17^ Our PAR approach involves collaboration with DWs and leaders of DW organisations to create a diverse and inclusive working group to formulate actions that are feasible and responsive to the needs of DWs.

The initial proposal was designed by a group of health and social researchers. Prior to its submission to the Women Rise initiative, it was presented to the major DW organisation in Lima, Peru. We asked women DWs for feedback to ensure we were responding to their needs. After receiving the funding, we presented the research proposal to a key department of the Ministry of Labour and Employment Promotion (Ministerio de Trabajo y Promoción del Empleo, in Spanish) and to each of the nine DW organisations registered in the Ministry of Labour.^18^ There are four DW’s unions and five independent training and advocacy organisations. Although no publicly available data exists on the proportion of DWs affiliated with an organisation, estimates suggest that membership is relatively low. Leadership typically consists of current or former DWs who are democratically elected. Some organisations have connections with international movements like the Latin American and Caribbean Confederation of Domestic Workers^19^ and the International Domestic Workers Federation.^20^

We used the research proposal as a reference point to build and negotiate the project structure, methods and outcomes. After a careful analysis of the different roles and power relations, it was decided to form two participatory committees.

The Co-researcher Committee includes active DWs who have previously participated in research projects. This group provides insights from their own or shared experiences and perspectives working as DWs. They help to build trust and ensure appropriate methods and representation. The co-researcher committee works together with the research team to ensure that the objectives and methods are aligned with the needs and interests of DWs. They participate in activities such as workshops on research methodology, identifying key informants, determining how and by whom they should be approached, playing an active role in developing data collection tools and evaluating their appropriateness. They attend scheduled meetings to discuss and provide input on the research, including what key information will be collected, how data will be collected and how results and reports will be shared.

The Advisory Committee includes current or former DWs who are leaders or representatives of DW organisations. By involving DW organisations representatives, we aim to improve understanding of the needs of DWs, increase ownership at both the individual and organisational levels, promote the feasibility of research procedures and support knowledge translation.

In addition to engaging with participants through our co-researcher committee and our advisory committee, we also engaged policymakers and academics as part of a steering committee.

The steering committee includes civil servants with a current or past role in key departments of public institutions responsible for ensuring formal working conditions for DWs, such as the Department of Fundamental Rights of the Ministry of Labour, the National Office of the Pension System, the National Superintendence of Labour Inspection and the Social Security. It also includes academics who have conducted research or implemented any programme related to DWs. The inclusion and engagement with members of the steering committee is progressive. This committee enhances the credibility of the study, the reports and the future recommendations. They play a key role in initiating policy discussions based on the findings.

**Figure 1:**
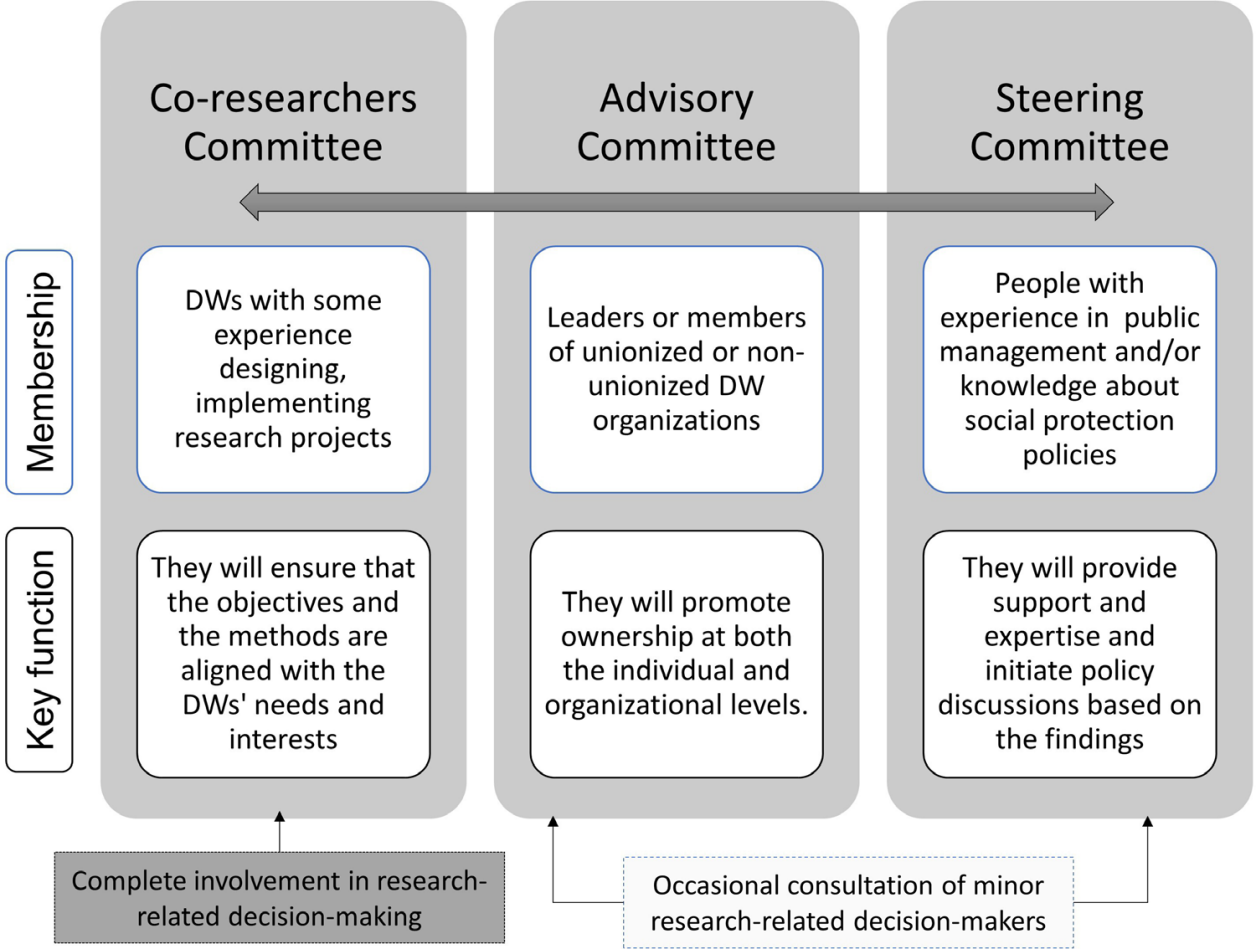
Characteristics of the members and key roles of the steering committee, the advisory committee and the co-researchers committee.

The differences in the members and key roles of the three committees are summarised in figure 1. Partnership and collaboration with the three committees is built through regular meetings, written updates and feedback by email or WhatsApp. Meetings are scheduled according to the availability of those attending. For the co-researcher and advisory committees, all organisations registered in the Ministry of Labour were contacted and invited to participate. We partnered with seven organisations, and they appointed two representatives (primary and alternate) to each PAR committee. Monetary incentives and snacks for attending each meeting are provided. The steering committee receives snacks for their participation, but no monetary incentive is offered because civil servants are not allowed to receive additional financial incentives.

## Methods

### Research design

**Figure 2:**
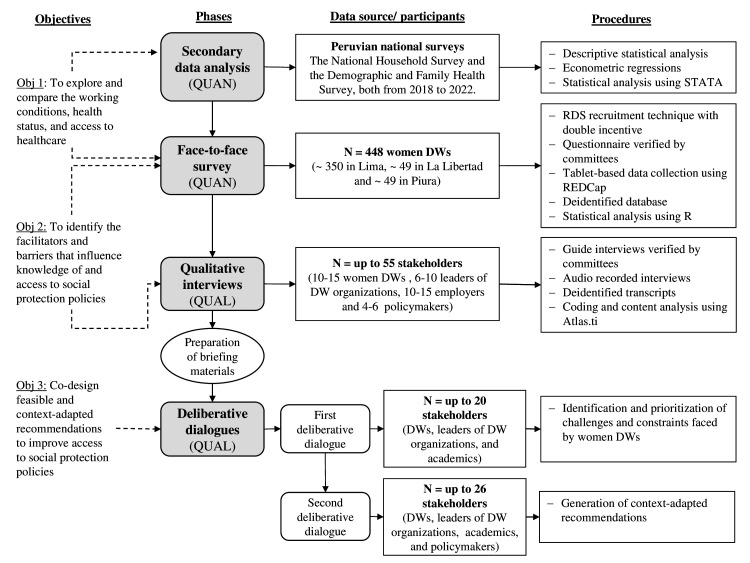
Sequential mixed-methods procedural diagram. DWs, domestic workers; QUAN, quantitative, QUAL, qualitative.

This study uses a sequential mixed-methods design^21^ structured in four phases: (1) secondary data analysis, (2) face-to-face survey, (3) qualitative interviews and (4) deliberative dialogues. We use mixed methods to build a comprehensive picture of the conditions in which DWs live, work and have access to social protection policies. Quantitative data will be analysed first to understand the constraints and challenges of social protection for DWs before and during the COVID-19 pandemic. The qualitative data will then deepen the quantitative findings by exploring in detail the experiences and attitudes of key informants. Finally, the quantitative and qualitative results will be integrated during data interpretation and used to initiate discussions in the deliberative dialogue sessions to develop recommendations for improving access to social protection for women DWs. All studies within the project will be conducted in Spanish. The phases of the project are outlined in figure 2.

For the face-to-face survey and qualitative interviews, data will be collected in three different cities in Peru with a high concentration of DWs: Lima (the capital), Piura and La Libertad (located on the northern coast). For 2020,^22^ 51% of DWs reported to live in Lima, 7% in Piura and 6% in La Libertad. We will describe and compare the characteristics of DWs in these three cities. We hypothesise that the working and living conditions may differ between the cities due to differences in geopolitical environment, culture and social values.

This project will be guided by two frameworks: the Social Determinants of Health^23^ (SDOH) developed by the WHO and the gender equity and socioeconomic inequality proposed by Moss.^24^ The SDOH framework addresses the interplay between social, economic and political mechanisms that shape determinants of health status. The Moss’ framework considers the geopolitical environment, culture, norms and sanctions, women’s roles, health-related mediators and health outcomes.

We are committed to promoting inclusivity across age, education, economic status, ethnic/racial self-identification, migration status and sector of work (formal and informal) to ensure the broadest possible range of participants. We aim to capture diversity and representation to provide rich and nuanced data to promote the visibility of historically marginalised and underrepresented groups.

The project was planned to run for 24 months in successive phases, starting from December 2022. At the time of manuscript submission, Phase 1 (December 2022 to April 2023) and Phase 2 (September 2023 to February 2024) had completed data collection. The planned start date for Phase 3 was April 2023, and the scheduled start date for Phase 4 was July 2024. Once data collection and analysis are complete, we plan to spend 2–4 months on dissemination activities.

### Phase 1: secondary data analysis (objective 1)

#### Data sources

We will use two Peruvian national surveys: the National Household Survey (Encuesta Nacional de Hogares, in Spanish), hereafter ENAHO, and the Demographic and Family Health Survey (Encuesta Demográfica y de Salud Familiar, in Spanish), hereafter ENDES. The ENAHO provides information about the living conditions, the evolution of poverty and the social support of the population. The ENDES provides data about demographics, health status and health-related behaviours. Both surveys are annual and use a nationally representative sample, including both urban and rural populations and are stratified to ensure representativeness at the national and regional levels. We will use the information from 2018 to 2022 to characterise and compare working conditions, health status and access to healthcare before, during and after the onset of COVID-19 in women DWs at the urban national level in Peru.

#### Data analysis

The ENAHO and ENDES databases are available to the public through the website of the Instituto Nacional de Estadística e Informática (INEI), in Spanish. A pooled dataset will be used, combining annual cross-sectional information from the National Household Survey (2018–2022) with information on those women who self-report working as DWs. The questions from the ENAHO and ENDES to be used for our analysis are listed in online supplemental material 1). The total sample includes 4216 DWs surveyed in their households over the 5-year period. Logit regressions will be used to identify the characteristics of DWs most affected by the pandemic, focusing on four outcome variables: (1) weekly hours worked (<48 hours/week and ≥48 hours/week), (2) hourly wage (<5 PEN/hour and ≥5 PEN/hour), (3) the presence of illness symptoms and (4) healthcare access. Control variables will include sociodemographic factors such as place of residence, self-identified ethnicity or race, education, age, poverty status, migrant status, health insurance coverage, number of children and marital status. Additionally, 3 year-specific dummy variables (for 2020, 2021 and 2022) and their interaction effects with each control variable will be included to determine whether the time moderation (ie, 2020 vs 2018–2019, 2021 vs 2018–2019, 2022 vs 2018–2019) for each characteristic is significant and whether its direction aligns with the main effect.

### Phase 2: face-to-face survey (objectives 1 and 2)

#### Participants

This phase consists of a quantitative cross-sectional survey of women DWs. We will include individuals older than 14 who self-identify as women and whose primary income is paid employment as DWs. We will not actively seek participants under 18, but since the ENAHO reports minors working as DWs, if we find potential participants under 18, they will be included in the study. We will include DWs who work full-time or part-time, are employed by a single household or through or by a service provider, are residing in the household of the employer (live-in) or are living in their own residence (live-out). To be eligible, they must have lived in Lima, La Libertad or Piura for more than 6 months. We will exclude women who do unpaid domestic work or do not do domestic work as their primary source of income.

#### Sample size and recruitment

We will use respondent-driven sampling (RDS),^25 26^ a network-based recruitment approach like snowball sampling that begins with a small convenience sample of individuals, known as ‘seeds’. It then propagates into recruitment waves, using peer referrals. RDS is best used to reach hard-to-reach groups, such as the DWs. RDS involves a dual incentive system, including a reward for participating and a reward for recruiting others into the study.

The sample size was calculated to detect an average of 40.4 hours per week based on the observed average working hours of DWs in ENAHO-2021. Considering an SD of 19.1 hours per week, a 95% confidence level and a desired total CI width of 5 units, we estimated an initial sample size of 224 participants.^27^ Balancing time, financial and human resource constraints, we assume an RDS design effect of 2, resulting in a planned sample size of n=448.

To promote a sample representative of the DWs’ population, we will recruit seeds with diverse age ranges, affiliation to DW organisation and division of labour (full-time vs part-time, or live-in vs live-out), as they are more likely to recruit participants with the same backgrounds. The co-researchers and advisory committee will identify seeds. We plan to recruit 12 seeds in Lima and 4 in La Libertad and Piura. Each participant will be limited to three peer referrals to encourage equal opportunity for referrals across all participants.

#### Procedures and data collection

We will design a survey that is sensitive to DWs and adapted to the Peruvian context, using validated questionnaires and questions from studies with similar populations. The survey will be organised into five domains: (1) individual and household characteristics, (2) working conditions, (3) health conditions, (4) healthcare access and (5) knowledge and perceptions about social protection policies. The variables in each domain are shown in table 1. The details of the measures and instruments can be found in online supplemental material 2. A data collection form will be created in Research Electronic Data Capture. Fieldworkers will be trained to use tablets to collect data. Training will include ethical considerations, gender sensitivity, RDS methods and psychosocial support.

**Table 1:** Domains and variables to be collected in Phase 2

Domain	Variable
1. Individual and household characteristics	Individual characteristics: gender self-identification, age, birthplace, marital status, union affiliation and social support.
	Household characteristics: education and literacy, ethnic/race self-identification, household location, migration history, digital connectivity access and usage, work-life balance, household composition, number of children, role distribution in the household, home ownership and social protection usage.
2. Working conditions	Time basis (full time/part-time), main employer’s characteristics, abuse experience, work environment, contractual situation, number of employers, working hours, wage, employment benefits, tasks and duties, home-to-work transportation cost and time, exposure to hazards, sickness absenteeism and presentism, rest break for medical issues, psychosocial risks and secondary job.
3. Health conditions	Anthropometric measures, blood pressure, health status, chronic health condition, COVID-19 history, depressive symptoms, lifestyle, sexual and reproductive health.
4. Healthcare access	Health seeking behaviour, utilisation of healthcare services, healthcare expenditure, access to social assistance/health insurance coverage, health literacy and social support.
5. Knowledge and perceptions about protection policies	Knowledge about Peruvian Domestic Workers’ law, breach of contract, union participation, perceived discrimination, knowledge about social protection, perceived working conditions and benefits.

We will seek expert judgement from the DW co-researchers and advisory committee members to ensure the survey’s appropriateness and ease of understanding. We will organise meetings with the co-researchers committee to review various instruments that measure the same constructs (eg, depressive symptoms using nine-item Patient Health Questionnaire or the Center of Epidemiologic Studies Depression Scale, 10-item version). After evaluating each instrument’s clarity, relevance and accessibility, the committee will determine the instrument that best aligns with the needs and experiences of DWs. To further refine the questionnaire, we will conduct a pilot with members of the advisory committee who will not participate in the study. Feedback from the pilot will allow us to clarify questions, adjust language and moderate the questionnaire length, ensuring that the final instrument will be both comprehensive and manageable for participants.

#### Data analysis

We will present simple estimates of counts/percentages for categorical variables, as well as means and SD or medians and IQR for continuous variables, depending on the distribution. The analysis will be done using the RDS package in R statistical software with RDS-II adjustment.^27^ It considers the weight of each participant based on their sampling probability according to their network size; DWs who are more connected to other DWs will receive a lower weight than those who are less connected. Later, we will conduct a post hoc sensitive analysis to assess the stability of the point estimates when seeds are removed.

### Phase 3: qualitative interviews (objective 2)

#### Participants

We aim to provide a nuanced understanding of the challenges and constraints of accessing social protection policies by gathering the perspectives and experiences of four groups of participants. (1) DWs: older than 18 years who self-identify as women, with at least 12 months of experience working as DWs within the last 5 years. (2) Leaders of DW organisations: both men and women, with at least 12 months of leadership experience at any given time, who may or may not be members of the PAR committees. (3) Employers: both men and women who coordinate the tasks of DWs and monitor their work performance, regardless of their role in hiring or remuneration. (4) Policymakers: civil servants with more than 12 months of working experience in the public sector and at least 3 months within the same department and/or institution. We expect to capture different narratives based on their distinct roles and positions.

The decision to conduct in-depth interviews rather than using other data collection methods is grounded in two key considerations. First, interviews allow us to capture the personal stories of DW and leaders of DW organisations, which is essential to understanding the unique experiences and challenges they face in both their daily lives and leadership roles. Conducting a focus group would not enable the same depth and authenticity in these individual narratives, as the group dynamic could limit the expression of nuanced personal and private experiences. Second, we opted for interviews with employers and civil servants due to the limited availability of time these participants typically have. From a logistical perspective, individual interviews were the most viable and practical option, enabling us to access their perspectives without compromising the quality or depth of the collected data.

#### Sample size and recruitment

We aim to interview individuals with diverse profiles and backgrounds for each group of participants. We estimate that we will interview between 10 and 15 DWs who work either full-time or part-time, in various arrangements including single households or service providers, living in or out of the employer’s household and from various age groups. Between 6 and 10 leaders of DW organisations at the national or local level, ideally one representative per organisation. Between 10 and 15 employers, including both those who report having a written or verbal employment contract. And, between four and six policymakers from key institutions are responsible for regularising, supervising or providing social protection policies. The final sample size for each group will depend on the saturation of the information collected.

We will use stakeholder mapping and feedback from the three committees to identify potential participants. We will recruit participants based on their accessibility and availability, as recommended by the co-researcher committee. We may also post invitations on social media to recruit employers. Some DWs interviewed may have also participated in Phase 2; however, we will not actively seek them out, as their identities will remain confidential. Additionally, we will carefully screen employers to ensure they have no previous or current relationship with any DW participants in this phase, minimising the risk of identification.

#### Procedures and data collection

The initial interview guides were developed by two research assistants and the two leaders overseeing this phase (JP and SPL), all with social sciences backgrounds and qualitative methods experience. The interview guides are structured in key themes, including access to social protection policies, perceptions of COVID-19 and its impact on the work of DWs and views on Peruvian legislation for domestic work. The interview guides can be found in online supplemental material 3). These themes are intended to further explore notable findings from Phases 1 and 2 by examining in detail the experiences and attitudes of the participants. To ensure all interview questions are sensitive and culturally appropriate, the guides will be shared with our advisory and steering committee members for review before data collection. This iterative process allows committee members to provide feedback, which the research team will then analyse thoroughly. Based on this input, the final version of the guides will be carefully edited to ensure they elicit meaningful, respectful and insightful responses from participants.

Data will be collected by experienced interviewers, such as sociologists, anthropologists or psychologists. Interviews will be audio-recorded. Written consent will be obtained before the interview begins. Interviews are expected to last about 1 hour, with about 20 questions. At the beginning of data collection, an initial analysis will be conducted after transcribing the first two interviews to refine the interview guide and ensure accurate coverage of themes. Interviews will be conducted online and in person, depending on participant availability. In Piura and La Libertad regions, all interviews will be conducted virtually due to the scarce availability of interviewers in those areas.

#### Data analysis

All interviews will be audio-recorded and transcribed verbatim. We will use a data extraction matrix for analysis. The matrix will allow us to make comparisons between actors and themes. The data analysis will be led by an expert in qualitative thematic analysis, supported by a trainee, with JP and SPL supervising the process as phase leaders.

We will adopt a thematic approach to analyse the data and complete the matrix, initially applying a pre-established set of codes derived from the SDOH^23^ and Moss.^24^ We will review and assess the consistency and agreement in coding practices across team members to ensure reliability. Through iterative review and discussions, codes will be adapted, and new codes may be introduced to capture emerging themes or nuances in the data.

### Phase 4: deliberative dialogues (objective 3)

#### Participants

Deliberative dialogues are a qualitative methodology that involves different stakeholders exchanging opinions on a particular topic to identify, clarify and align expectations among participants.^28 29^ We will have two consecutive deliberative dialogues, the first with DWs, leaders of DW organisations and academics. The second dialogue will also include policymakers from the Ministry of Labour, the Social Security and others. The first dialogue aims to identify and agree on 1–3 barriers per thematic axis (working conditions, health status and access to healthcare services). The second dialogue aims to generate recommendations to overcome these barriers.

#### Sample size and recruitment

For the first deliberative dialogue, we anticipate 14–20 participants. Diversity will be ensured by having representatives from each DW organisation from the three committees and DWs not affiliated with any organisation to reflect various working conditions and residences. We will include academics with expertise in DWs working conditions and labour rights research. For the second dialogue, we estimate between 18 and 26 participants. We will invite participants from the first deliberative plus policymakers. We will recruit participants by snowballing, using the different networks of the three committees as starting points.

#### Procedures and data collection

Before the deliberative dialogues, we will prepare and disseminate a knowledge brief to participants that provides background information to encourage thoughtful and informed discussions. The materials will be visual or audiovisual and based on the key findings from phases one through three. An expert facilitator will moderate both dialogues and train members of the research team to facilitate small group work.

Participants will be divided into three groups: DWs, leaders of DW organisations and academics. A research team member will be assigned to each group to function as a facilitator. First, each group will review the previously shared briefing materials, clarify any questions and create a group summary. Second, each group will identify, reflect and prioritise challenges and constraints by thematic axis (working conditions, health status and access to healthcare services).

There will be a 4-week gap between the first and second deliberative dialogues. This period will allow the participants to reflect and will allow the research team to summarise the results of the first deliberative dialogue. For the second deliberative dialogue, the primary facilitator will guide the session. First, the facilitator will present the summary of findings from the first deliberative dialogue prepared by the research team and clarify any questions. Second, each group that participated in the first dialogue will present the challenges and constraints they agreed on. They will be able to make additions or changes if the group perspective has changed. Third, policymakers will be asked to provide their perspectives, focusing on issues related to their field. Finally, all the participants will be asked to deliberate until a consensus is reached and to work together to develop two or three recommendations per thematic axis.

To minimise any potential power imbalances among participants, facilitators will be briefed to actively encourage contributions from all attendees, paying particular attention to the voices of individuals who may feel less empowered, such as DWs. By setting ground rules for respectful and inclusive dialogue at the beginning of the session, we aim to create a safe environment where all participants feel valued and respected, regardless of their role or occupation.

#### Data analysis

While no transcription is planned, recordings and field notes will serve as resources to capture all pertinent points and notable quotes. The plenary discussions will be summarised in a comprehensive report detailing the various perspectives, arguments and proposals presented during the deliberative dialogues. This report will serve as the basis for the preparation of dissemination materials to communicate the recommendations with a broader audience.

### Ethics and dissemination

#### Ethics

Phases 1 and 2 received ethical clearance from Universidad Peruana Cayetano Heredia in Peru (#544-48-22 and #286-21-23) and Unity Health Toronto in Canada (REB #23–196). Phases 3 and 4 received ethical clearance from PRISMA Charitable Association in Peru (#0127–24) and Unity Health Toronto in Canada (#24–080).

The informed consent forms were designed to be culturally sensitive and accessible, considering the diverse backgrounds and literacy levels of DWs. Participants will be assured of their right to withdraw from the study without repercussion, thereby prioritising their autonomy and agency throughout the research process. Individuals with reading difficulties (eg, due to low literacy or poor sight) may ask for assistance from a third party of their choice to be with them while the consent documents are read to them. However, once the consent is provided, the third party will be asked to leave the interview venue to ensure the privacy and comfort of the consenting participants. The research staff will be attentive to prevent possible co-action by the third party that provides assistance.

DW participants younger than 18 years will also be asked to provide written informed consent. Recently published ethical considerations for research with adolescents highlight how they should be viewed as individuals with their own personhood, as evidence shows they think similarly to young adults.^30^ We believe that the survey can make adolescents working as DWs visible and help to understand their particular conditions and needs, so their inclusion in the study is important. Additionally, adolescents who work often develop a level of maturity and understanding, equipping them with the capacity to provide informed consent for research that may benefit them.^31^ Their real-world experiences and responsibility can enhance their ability to comprehend the research process, potential risks, and benefits involved.^31^ We will encourage adolescents to seek advice from their parents or other trusted adults where appropriate. We will prioritise staff with previous adolescent health or well-being experience to obtain informed consent.

Although this study poses no direct harm, participants may feel uncomfortable discussing their work or health conditions. To address this, they will have the option to skip any questions they prefer not to answer. Signs of trauma or violence may emerge during face-to-face surveys or interviews. In such cases, a protocol will be in place to connect interviewees with appropriate support services. Research staff will be trained in emotional support and active listening but will not serve as counsellors or therapists. They will also be prepared to pause interviews to safeguard participants’ well-being. The team will provide brochures with contact information for institutions that provide physical, emotional or social support and information about DW’s labour rights.

The ANITA project is committed to protecting the privacy and confidentiality of participant data through strict data management practices. Databases and transcripts will not contain identifiers to preserve the anonymity of participants. Audio recordings will be deleted after transcription. Physical documents such as informed consents will be kept in a locked cabinet, while digital materials such as transcripts or databases will be stored on a secure, password-protected server at the Universidad Peruana Cayetano Heredia. Only the research team will have access to the information in both cases.

### Dissemination

The research team will produce dissemination outputs for internal team meetings, workshops in deliberative dialogues and peer-reviewed journals. We will work with our PAR committees to ensure dissemination strategies meet their needs and channels of communication. This may include leveraging social media (eg, Twitter and Facebook) and non-traditional media of the partner organisations and their newsletters to share news and outputs (eg, pictorial storyboards). Special efforts will be put into sharing the results with the stakeholders who participated in deliberative dialogues. During the workshops, we will also include training on advocacy techniques to empower DWs and their leaders. Finally, we will generate policy briefs and videos to share the policy recommendations with different audiences and sectors.

The knowledge mobilisation strategies of the ANITA project will have three overall objectives: (1) to engage relevant stakeholders in all project activities over the 2-year collaboration; (2) to sustain equitable relationships between the project partners and the Women RISE Administration and (3) to disseminate project activities and research outputs and findings to relevant publics via various communication channels.

At the end of the project, three workshops will be organised at each study site (Lima, La Libertad and Piura) to thank the participants for their involvement and to present the main results and outputs of the project. We will invite the women DWs who participate during data collection and public officers from the regional and national departments of health, labor and social security.

### Reflexivity

For the ANITA project, having a collaborative and respectful relationship with DWs is central. This was considered since the formulation of the research project proposal. For this reason, we carefully chose the acronym ANITA. Aside from borrowing key letters from the title, ANITA is also the diminutive form of a common Peruvian woman’s name. It is common for employers to call DWs by their diminutive names to express affection and family integration. Our project uses the name ANITA to convey positive feelings and respect for DWs.

To ensure project success, our multidisciplinary team will leverage expertise across a wide range of disciplines, including health and medicine, economy, social sciences, epidemiology and public health. JTM, JP, MLP, MKC, SPL, NAP and MSCF have individually and collectively taken part in research projects with an emphasis on chronic disease, mental health and women’s health. In 2020, JTM, JP and MLP developed a study that explored violence against women in emergency contexts and produced recommendations for civil servants to better manage these situations. APH, CM and ADP recently completed the EMPOWER research project, which examined the work conditions and health of personal support workers (PSWs) in Toronto prior to and during the COVID-19 pandemic.^32 33^ There are many similarities between the PSWs population in Canada (ie, recent immigrants and racialised, low-income women facing precarious employment) and DWs in Peru. NAP has more than 13 years of work experience across different sectors in gender equality at work. DVT and VC have experience coordinating impact assessments of public programmes and policy analysis with international cooperation agencies and multilateral organisations. Each member has actively and intellectually contributed to developing the research proposal and is fully committed to carrying out the relevant tasks from inception to disseminating knowledge and recommendations from the project.

Though challenging, we believe that PAR promotes DWs empowerment and equitable collaboration. It also may enable future collaborative research and policy generation. Not only will members of the three committees participate in the project’s activities, but we will also actively participate in events and initiatives they organise (eg, forums or webinars). The involvement of other academics who are also researching DW will allow us to strengthen our efforts, achieve comprehensive results and promote interinstitutional collaborative work. Additionally, researchers from other countries can replicate the approach used in this project to foster collaborations between academia, decision-makers and other stakeholders.

## supplementary material

10.1136/bmjopen-2024-088921 online supplemental file 1

10.1136/bmjopen-2024-088921 online supplemental file 2

10.1136/bmjopen-2024-088921 online supplemental file 3
