# Supplementary material for: Addressing the challenges and constraints of social protection policies for Peruvian women domestic workers: the ANITA project study protocol
Source: BMJ Open. 2025 Mar 6;15(3):e088921. doi: 10.1136/bmjopen-2024-088921 (PMC11887307; doi:10.1136/bmjopen-2024-088921)
Supplement: online supplemental file 2 [file bmjopen-15-3-s002.docx]

**Supplementary material 2: Summary of the instruments to be used for data collection according to the domains and variables of interest.**

| *Domain* | *Variables* | *Instrument* | *Adequate for DWs?* |
| --- | --- | --- | --- |
| i. Individual and household’s characteristics | Household characteristics:   - Household composition - Household location - Number of children - Role distribution in the household - Home ownership     Individual’s characteristics:   - Age - Birthplace - Marital status - Education and literacy - Digital connectivity access and usage - Ethnic/ race self-identification - Religion - Migration history - Social support | The Peruvian national household survey (ENAHO)^[1]^. It targets people above aged 13. | Yes, the ENAHO has been designed to be applied in domestic workers living in the house they work at regardless if they receive a compensation for their work or not. |
|  |  | The Peruvian Working Conditions, Safety and Health Survey (CENSOPAS) ^[2]^  It targets people above aged 13. | Yes, the CENSOPAS survey has been designed to be applied in domestic workers living in or outside the house they work at. |
|  |  | The multidimensional scale of social support is a 12-items measure of perceived adequacy of social support from three sources: family, friends and significant other, using a 5-point Likert scale.^[3]^ | Yes, the Spanish version has been validated in vulnerable migrant DWs population^[4]^ |
| ii. Working conditions | - Wage - Contractual situation - Time basis (Full time/part-time) - Number of employers - Main employer’s characteristics - Work environment - Employment benefits - Tasks and duties - Exposure to hazards - Psychosocial risks - Abuse experience - Home-to-work transportation cost and time - Secondary job - Work-life balance | The Peruvian Working Conditions, Safety and Health Survey (CENSOPAS) ^[2]^  It targets people above aged 13. | Yes, the CENSOPAS survey has been designed to be applied in domestic workers living in or outside the house they work at. |
|  |  | The European Working Conditions Survey (EWCS) provide detailed information on a broad range of issues, including exposure to physical and psychosocial risks, work organization, work–life balance, and health and well-being. It targets people above aged 15. ^[5]^ | Yes, the EWCS survey has been designed to be applied in residents in employment at the time of the survey, including domestic workers living in or outside the house they work at. The Spanish version has been validated widespread and used in a previous research study on DWs from Argentina but not in Peru. ^[6]^ |
| iii. Health conditions | - Anthropometric measures - Health status - Blood pressure - Depressive symptoms - Lifestyles - Chronic health conditions - Sexual and reproductive health - COVID-19 history | The 12-Item Short-Form Survey (SF-12) a 5-point Likert scale.  Or  The EQ-5D-5L questionnaire measures health-related quality of life and contains 5 questions to be answered on a Likert scale and one question on an analogous scale from 0 to 100.    Both questionnaires have demonstrated good measurement properties and good construct construction ^[7,8]^. | The SF-12 and the EQ-5D-5L both have a widespread use and validation in Spanish and in Peruvian adult and vulnerable population but not in DWs.    ****We will test the feasibility of both questionnaires with the co-researchers committee. They will help us to decide the most suitable for the DWs population.*** |
|  |  | The Center for Epidemiologic Studies Depression Scale (CES-D-10), a 10-item self-report measure of occurrence of depressive symptoms *“during the past week”* that uses a 5-point Likert scale  Or  The Patient Health Questionnaire (PHQ-8) rates the frequency of symptoms over the past 2 weeks on a 0-3 Likert-type scale to assess depression severity (from minimal depression to severe depression).^[9]^ | The CES-D-10 and the PHQ-8 both have a widespread use and validation in Spanish and in Peruvian adult and vulnerable population but not in DWs.  ****We will test the feasibility of both questionnaires with the co-researchers committee. They will help us to decide the most suitable for the DWs population.*** |
|  |  | The Pandemic Stress Index, a is a 3-item measure of behavior changes and stress that individuals may have experienced during COVID-19. ^[10]^ | The Spanish version has been validated in vulnerable migrant adult population^[11]^ but not in DWs.  ****We will test the feasibility of both questionnaires with the co-researchers committee. They will help us to decide the most suitable for the DWs population.*** |
| iv. Health care access | - Sickness absenteeism and presenteeism - Rest break for medical issues - Healthcare expenditure - Access to social assistance/ health insurance coverage - Utilization of health care services - Health seeking behavior - Health literacy | The U.S. Health Information- National Trends Survey (HINTS) to investigate respondents’ access to and use of health information ^[12,13]^ | The Spanish version has been validated in vulnerable migrant adult population but not in DWs. ^[14]^  ****We will test the feasibility of both questionnaires with the co-researchers committee. They will help us to decide the most suitable for the DWs population.*** |
|  |  | The three Health Literacy Screening questions to detect inadequate health literacy among adult patient populations.^[15]^ | The Spanish version has been validated in vulnerable migrant adult population but not in DWs.^[16]^  ****We will test the feasibility of both questionnaires with the co-researchers committee. They will help us to decide the most suitable for the DWs population.*** |
| v. Knowledge and perceptions about protection policies | - Knowledge about Peruvian DW’s law - Union affiliation - Work autonomy - Work related satisfaction.      - Perceived discrimination | The *items related to knowledge about Peruvian DWs are of research team´s own creation.* | * All the items will be co-design with the co-researchers committee. |
|  |  | The Everyday Discrimination Scale (short version), a 5-items scale to evaluate experiences of perceived discrimination.^[17]^ | The Spanish version has been validated in vulnerable migrant DWs population^[4]^ but not in Peru.  ****We will test the feasibility of both questionnaires with the co-researchers committee. They will help us to decide the most suitable for the DWs population.*** |

**References:**

1. ENAHO. Encuesta nacional de hogares 2021. 2021;1–25.

2. Condiciones de Trabajo y Salud – CINDOC – CENSOPAS [Internet]. [cited 2023 Apr]. Available from: https://censopascindoc.ins.gob.pe/tag/condiciones-de-trabajo-y-salud/

3. Multidimensional Scale of Perceived Social Support (MSPSS) | Measures Library [Internet]. [cited 2023 Mar]. Available from: https://elcentro.sonhs.miami.edu/research/measures-library/mspss/index.html

4. Kim Y, Lee H, Lee M. Social Support for Acculturative Stress, Job Stress, and Perceived Discrimination Among Migrant Workers Moderates COVID-19 Pandemic Depression. Int J Public Health [Internet]. 2022 [cited 2023 Mar];67. Available from: /pmc/articles/PMC9413060/

5. Sixth European Working Conditions Survey: 2015 | Eurofound [Internet]. [cited 2023 Apr]. Available from: https://www.eurofound.europa.eu/surveys/european-working-conditions-surveys/sixth-european-working-conditions-survey-2015

6. Fernanda Bauleo M, Van Dijk F, Radon K. One’s workplace, other’s home? Work and health of domestic workers in Argentina. Ann Glob Health. 2018;84(3):450–8.

7. Johnson JA, Coons SJ. Comparison of the EQ-5D and SF-12 in an adult US sample. Qual Life Res [Internet]. 1998 [cited 2023 Mar];7(2):155–66. Available from: http://www.ncbi.nlm.nih.gov/pubmed/9523497

8. Johnson JA, Pickard AS. Comparison of the EQ-5D and SF-12 health surveys in a general population survey in Alberta, Canada. Med Care [Internet]. 2000 [cited 2023 Mar];38(1):115–21. Available from: https://pubmed.ncbi.nlm.nih.gov/10630726/

9. PHQ-8 described in ePROVIDE [Internet]. [cited 2023 Apr]. Available from: https://eprovide.mapi-trust.org/instruments/patient-health-questionnaire-8-items

10. The Pandemic Stress Index (PSI) [Internet]. [cited 2023 Mar]. Available from: https://elcentro.sonhs.miami.edu/research/measures-library/psi/index.html

11. Kim Y, Lee H, Lee M. Social Support for Acculturative Stress, Job Stress, and Perceived Discrimination Among Migrant Workers Moderates COVID-19 Pandemic Depression. Int J Public Health. 2022;67:151.

12. Cancer Institute N. HINTS , Cycle 3, Full Content English Instrument, Annotated.

13. Survey Instruments | HINTS [Internet]. [cited 2023 Mar]. Available from: https://hints.cancer.gov/data/survey-instruments.aspx#H5C3

14. Pena-y-Lillo M. A profile of the health information seeker in Chile: Introducing the Chilean health information environments (EIS) survey. World Med Health Policy [Internet]. 2022 [cited 2023 May];14(2):295–309. Available from: https://onlinelibrary.wiley.com/doi/full/10.1002/wmh3.486

15. Health Literacy Questions | Measures Library [Internet]. [cited 2023 Mar]. Available from: https://elcentro.sonhs.miami.edu/research/measures-library/health-literacy-questions/index.html

16. Sarkar U, Schillinger D, López A, Sudore R. Validation of Self-Reported Health Literacy Questions Among Diverse English and Spanish-Speaking Populations. J Gen Intern Med [Internet]. 2011 [cited 2023 May];26(3):265. Available from: /pmc/articles/PMC3043178/

17. Everyday Discrimination Scale | David R. Williams [Internet]. [cited 2023 Mar]. Available from: https://scholar.harvard.edu/davidrwilliams/node/32397
