# Supplementary material for: Addressing the challenges and constraints of social protection policies for Peruvian women domestic workers: the ANITA project study protocol
Source: BMJ Open. 2025 Mar 6;15(3):e088921. doi: 10.1136/bmjopen-2024-088921 (PMC11887307; doi:10.1136/bmjopen-2024-088921)
Supplement: online supplemental file 3 [file bmjopen-15-3-s003.pdf]

ANITA Project – Phase III

Interview Guide for Domestic Workers (DWs)

I. SOCIODEMOGRAPHIC DATA

1. What city/district do you live in?
2. In which city/district do you currently work?
3. How long does it take you to travel from your home to your workplace?
4. How old are you?
5. What is your marital status?
6. What is your highest level of education?
7. According to your customs and/or your ancestors, how do you identify yourself ethnically? (e.g.: mestizo, Afro-Peruvian, Andean, Amazonian, etc.)
8. How long have you been working as a DW?

   ***Inquire**: if you have had other different jobs.*

9. Are you currently involved in any DWs movement, organization or union?

   In-depth: reasons, implications that your affiliation has generated in your work

II. LABOR TRAJECTORY

10. Since when did you start working as a DW? Why?
    ***In-depth**: Age, context, reasons, time working in the field.*
11. How do you feel about working as a DW?
    ***In-depth:** emphasize how housework makes you feel: how did you feel about it? beginning and how this perception changed over time.*
12. Some employers often say that DW who work in their home are part of their family. What do you think about this?

III. WORKING CONDITIONS

13. What tasks do you perform at your workplace?
    ***Inquire**: Whether or not you think it is your responsibility to do these tasks and why.*
14. What agreements do you have with your employer?
    a) What are your working hours?
    b) Live-in work or live-out work?
    c) Are the activities you mentioned above the same as those you initially agreed upon with your employer?
    d) What days do you work? What days do you rest?
    e) Do you wear a uniform at work??

In-depth*: Perception about the use of the uniform*

15. What type of employment contract do you currently have with your employer? What do you think of this type of contract?

    Inquire: *What additional benefits do you have outside of those established by the Law 31047 (e.g. vacation, bonus, compensation, pension by AFP or ONP)*

    For DW without a contract, inquire: *Have you tried to talk to your employer about it? Do you know what the law is?*

IV. HEALTH CONDITIONS

16. If you feel any discomfort, what do you do? Where do you go? Why?
    Inquire: *If feeling this way affects your work*
17. What do you do to take care of your health?
    In-depth: *If you use traditional medicine, nutrition, exercise.*
18. What do you do to take care of your mental health?
    Inquire*: Mood, motivation, emotional support network (who do you turn to) when you have a personal problem or to ask for advice).*
19. What aspects would limit you from seeking care at a health center?
    Inquire*: priorities for your health care, if you are dedicated to caring for others at home, costs, distance from your home to the center, time to access consultation.*
20. Do you have health insurance?

    IF SHE HAS health insurance:

    a) What type of health insurance do you currently have?
    b) Do you use your health insurance? Why?
    c) Do you have any difficulties using health insurance? Which ones?
    Inquire: frequency of use, whether you like it or not, how long you have had it.
    d) Who pays for your insurance?

    SHE DOES NOT HAVE health insurance:

    e) Why don't you have health insurance?
    f) Would you like to have health insurance? Has your employer offered you health insurance?
    g) Are you aware of the benefits of receiving health insurance?

21. Have you ever had an accident during your work day?
    Inquire*: what was the care procedure, attitude of the employer.*
22. Have you ever worked while feeling unwell/sick?
    Inquire if yes*: If you were given permission to be absent, how did you act? employer.*
23. Have you ever worked as a DW while pregnant? How did you handle this situation?
    Inquire*: about the experience, complications at work due to pregnancy, if you had leave of absence or maternity leave before and after childbirth, if she received maternity leave under what conditions, decision of delay pregnancy due to the implications for their work.*

24. **Only for DWs who are mothers,** What kind of flexibility has your employer given you for activities that involve caring for your child? (medical appointments, school activities)

V. COVID-19

Now we want to ask you about the time of the pandemic and how it influenced your role as DW.

25. Did you continue working during the pandemic period?
26. Have you changed your work modality (live-in or live-out work)?
27. Have there been any changes in the dynamics of your work? (tasks, working hours, salary)
28. What preventive measures did you take at your workplace?
29. Were there any special measures for you? Which ones? How did you feel?
30. Did you receive any support from the State? (Economic bonuses such as Yanapay, Bono 600, Universal Family Bonus)
31. Have you received any extraordinary support from your employer during the COVID-19 pandemic? What kind?
32. Did you get sick with COVID-19 during that time?
    Inquire*: actions of your employer when the DW became ill, how the DW felt with it.*

VI. KNOWLEDGE AND PERCEPTIONS ON SOCIAL PROTECTION POLICIES (LAW 31047)

33. What rights do you know that DWs have?
    Inquire*: If your employer knows about the rights of DWs, where is it reported, where you can learn about your rights.*
34. Have you ever suddenly found yourself without job? What happened?
    Inquire*: whether she was fired or why she resigned.*
35. Did you have savings or received any compensation or severance pay? How did you cope with the unexpected changes during this period?
36. Are you familiar with Law 31047, the law on domestic workers?
    If you answer YES:
    a) Do you know when it was approved?
    b) What is it about?
    c) What are your expectations with this law?

    If you answer "NO":

    d) Do you think that domestic workers need a law?
    e) What should this law have?
37. Who do you think should monitor the rights of domestic workers?
38. What do you think are the responsibilities of the employer and the responsibilities of the employee for compliance with the rights of DWs?
39. Finally, is there anything else you would like to add that has not been mentioned in the previous questions?

ANITA Project – Phase III

Interview Guide for Domestic Workers Leaders [i]

I. SOCIODEMOGRAPHIC DATA

1. What city/district do you live in?
2. How old are you?
3. What is your marital status?
4. What is the highest level of education you have?
5. According to your customs and those of your ancestors, how do you identify yourself ethnically? (e.g.: mestizo, white, Afro-Peruvian, Andean, Amazonian, etc.)
6. What are you currently doing?

   ***Indication**: The way you name your profession can be used around the interview instead of DWs.*

7. If you work as a DWs, how many years have you been working as a DWs?
   ***Inquire**: if you have had other different jobs.*

II. LEADERSHIP TRAJECTORY

8. To which organization do you belong to?
9. How long have you been a member of this organization?
10. At what stage did you decide to join the organization?
    ***Inquire:** whether the employer has prevented or hindered you from joining a union or exercising your right to work her position*
11. What position do you hold within this organization? Since when?
12. How long has the organization existed and how many DWs are affiliated?
13. What is the purpose of the organization?

III. WORKING CONDITIONS OF DWs

14. Based on your experience, what is the most common type of contract that DWs have? Why?
15. What rights do you know that DWs have?

    ***Inquire**: Compensation for job service, settlement and retirement.*

16. What are the main labor rights with less access by DWs? Instruction: For each one, mention at least one barrier/cause of that lesser access. *access.*
    ***Inquire:** 3 main rights*
17. Which groups of DWs have less access to their labor rights? Why do you think this is?
    ***Inquire:** Migrants, adolescents and minors, older adults, Afro-Peruvians, trans women, Andean-Amazonian, etc.*
18. Based on your experience, what barriers exist to formalizing housework?
    ***Inquire**: other factors that can facilitate this process, political will of the authorities, expand the movement of DWs, etc.*

19. From your role as a leader, could you tell us what are the main demands of DWs regarding their employment situation?
20. Some employers often say that DWs who work in their home are part of their family. What do you think about this?
21. Do you think that housework is valued by society? Why?

IV. HEALTH CONDITIONS

22. What are the main health problems of DWs? Why?
    Inquire: *Health problems at work, accidents at work, sick leave maternity*
23. When a DW has a health problem, where do they usually go? Why?
24. Do you think most DWs have health insurance?
25. What are the main difficulties that DWs have in accessing health insurance?

V. COVID-19

Now we want to ask you about the time of the pandemic and how it influenced the work of the DWs.

26. What were the main challenges faced by DWs during the pandemic?
27. What were the consequences of these problems on the daily lives of DWs?
28. By 2024, do you think any of these problems will persist? Which ones and why?

VI. KNOWLEDGE AND PERCEPTIONS ON SOCIAL PROTECTION POLICIES(law 31047)

29. Do you know Law 31047, the Domestic Workers Law?
    a) Do you know when it was approved?
    b) What is it about?
    c) What are your expectations with this law?
30. In your experience, what are the gaps you have found in the law so far?
31. What actions should the State take to comply with Law 31047?
    Inquire: *MTPE, SUNAFIL, MIMP.*
32. What do you think is your organization's role in compliance with Law 31047?
33. What actions does your organization take to comply with Law 31047?
    Inquire: *on resources and/or needs to carry out their actions as organization*

ANITA Project – Phase III

Interview Guide for Domestic Workers' Employers

I. SOCIODEMOGRAPHIC DATA

1. What city/district do you live in?
2. How old are you?
3. What is your marital status?
4. What is the highest level of education you have?
5. According to your customs and/or your ancestors, how do you identify yourself ethnically? (e.g.: mestizo, Afro-Peruvian, Andean, Amazonian, etc.)
6. What are you currently doing?
7. Who lives with you at home? Who is the head of your household?

II. WORKING EXPERIENCES WITH DWs

For each adult living in the household, ask: Are they currently working? Under what modality (in-person, remote, hybrid)? Full-time/part-time?

8. How long have you been hiring DWs? (generally, this is counted from the first time you hired a DW for your home)
9. Since that year, to date, how many DW have you hired?
   Inquire*: Reasons for resignation and/or dismissal from DW*
10. During your childhood or youth, did you have the services of a DW in your home?
11. Some employers often say that DW who work in their home are part of their family. What do you think about this?
12. How many DWs currently work in your home? Under what regime? (With residence/without residence) If there is more than one DW, you could write down their names and the questions from here on would be for each one.
    Inquire*: if you have DW with residence, contract agreements with residence (benefits, where in the house they live)*
    In-depth: *If it is live-in work, more information about where they live…what part of the house...what benefits do they have (bed, TV, window, water) hot, etc.).*
13. How long have you been working with the DW who works in your home?
14. How did you come to contact her?
    Inquire*: If I use an employment agency or by reference, Facebook, friends, relatives.*
15. Are there any requirements when hiring the services of a DW? Why?
16. What tasks does the DW perform in your home?
    Inquire*: If you cook, wash, iron, take care of children, take care of pets, etc.*

17. What kind of rules or practices to follow have you established with the work of the DW in your home? (Use of uniform, How many hours a week does the DW work – weekly average, space where you eat breakfast/lunch/dinner, cell phone use)

III. WORKING CONDITIONS

18. What type of agreement or contract do you have with DW?
    a. How was the process for making the agreement or contract?
    b. What difficulties did you encounter during the process of negotiating the agreement or contract?
19. What formal benefits does DW have?
    Inquire: Health insurance, AFP, bonus, Compensation for job service, vacations, breaks, holidays *maternity/sick leave.*
20. Do you provide any additional benefits/incentives to the DWs salary? (such as clothing, food, groceries, food vouchers in supermarkets, support with their children, payment for studies, etc.) Why?
21. Do you provide any kind of health insurance? *(EsSalud, SIS or Private Insurance)*to DW? Why?

IV. HEALTH CONDITIONS

22. What happens when the DWs suffers from a health problem?

    Inquire*: if they give you permission or medical leave, if they support you with your medicines, who supplies lhousehold chores, how they manage time to go to the doctor - do they do it? Do they give another day as a vacation day, or do they deduct it?*

23. Do you know where your DW usually gets care?

    Inquire: *If the DW uses the DWE health insurance, if he/she goes to the hospital or clinic, ifis not attentive to the health status of the DW.*

24. Has a DW ever worked for you while you were pregnant? How did you handle this situation?
    Inquire*: about the experience, complications at work due to pregnancy, if you were given leave of absence or maternity leave before and after childbirth, whether the DW received maternity leave and under what conditions.*

V. COVID-19

Now we want to ask you about the time of the pandemic and how it influenced your working relationship with the DW.

25. During the pandemic, did you work with the same DW that you work with now? Why is it still/no longer?

26. During the pandemic, some employers asked the DW to live with them. How was your experience and what implications (regarding the employment relationship and emotional bond) did it have on your relationship with your DW?

    ***Inquire**: amount of DWs in the home, experiences with dismissal during the pandemic.*

27. Did the DW have any special protection measures? (use of mask, protective suit, gloves) Why was that?

28. Did you provide any exceptional support to DW during this period?

    ***Inquire:** Payment for PCR tests, transportation in taxis to avoid contagion, medications for COVID symptoms, financial support, etc.*

VI. KNOWLEDGE AND PERCEPTIONS ON SOCIAL PROTECTION POLICIES (LAW 31047)

29. Do you know what duties the employer has with regard to the DW? What do you think they are?

    ***Inquire:** Bonus, health insurance, AFP, CTS, paid vacations, etc. where you were informed, where you can learn about your duties.*

Now we would like to ask you about the new Law on domestic workers.

30. Have you heard about this law?

    <u>If you know</u> of the law:

    a. How did you find out?
    b. What is your opinion on this law?

    <u>Does not know</u>of the law:

    a. What rights do you think DWs have?
    b. Who do you think is the authority responsible for enforcing this new law?

31. What facilities should the Peruvian State provide to promote the formalization of domestic work?
32. Based on your experience as an employer, what recommendations could you provide so that other employers can adequately comply with the labor rights of DWS?
33. What is your opinion regarding the work of DWS and their role in home care?

ANITA Project – Phase III

Interview guide for Decision-Makers

I. **SOCIODEMOGRAPHIC DATA**

1. What district do you live in?
2. How old are you?
3. What is your marital status?
4. What is the highest level of education you have?
5. According to your customs and/or your ancestors, how do you identify yourself ethnically? (e.g.: mestizo, Afro-Peruvian, Andean, Amazonian, etc.)
6. Where do you currently work?
7. What position do you hold?

I. OPINIONS ON THE SITUATION OF DWs IN PERU

8. In general terms, what do you think of the changes introduced by Law 31047 “Law of domestic workers” that was approved in October 2020 in reference to the rights of DWs? Are there aspects that you consider outstanding (positively or negatively) regarding this Law? Could you tell me which ones and why you consider them this way?
9. If we compare this Law with its previous regulations, what are the main changes or differences that you see between them? Which do you consider to be the most significant changes? Why? From your perspective, do these changes or differences represent improvements or setbacks in the regulations? Why?
10. From your experience/point of view, do you think that DWs in Peru can fully exercise their rights under the law? Why?

    If the interviewee has difficulty answering, give as an example; having an employment contract, insurance with EsSalud, access to the pension system, maximum hours worked per week, etc.

11. Do you identify or perceive differences between DWs in Lima and DWs working in other regions in relation to the full exercise of their rights? Do you know of other differences between DWs associated with age, ethnicity or other factors that could generate inequalities among this same group in Peru?

II. BARRIERS AND LIMITATIONS THAT AFFECT THE RIGHTS OF DWs SPECIFIC TO THE SECTOR/ENTITY OF THE DECISION-MAKER INTERVIEWED

12. Based on your experience, what are the main barriers and limitations that DWs have in accessing the full exercise of their rights related to their working and health conditions?

In-depth: Delve deeper into the topic that is linked to the interviewee's sector and investigate whether differences are perceived by region and/or identity (gender, ethnicity).

13. Why do you think these problems occur? For each barrier mentioned in the previous question, ask them to identify at least one or two causes and elaborate on each one.
14. Do you think there are any measures that could be implemented to address these problems?

III. SECTOR/ENTITY-SPECIFIC INITIATIVES OF THE INTERVIEWED DECISION-MAKER FOR THE BENEFIT OF DWs

15. From your sector, what initiatives or projects have been carried out that directly or indirectly involve DWs? Ask for more information on each of them (scope, objective, budget, progress in implementation, bottlenecks, etc.)
16. Are any initiatives in your sector being planned or implemented to support domestic workers? Ask for more information on each of them (scope, objective, budget, progress in implementation, bottlenecks, etc.)
17. <u>For the members of the Multisectoral Working Group</u>: What do you consider to have been the main results or products of this working group?

IV. COVID-19

18. What do you think were the main problems that DWs faced during the pandemic?
19. From your sector, was there any type of support or collaboration with DWs and/or their organizations to mitigate the effects of these problems?
20. By 2024, do you think these problems will persist? Why?
