# Supplementary material for: Addressing the challenges and constraints of social protection policies for Peruvian women domestic workers: the ANITA project study protocol
Source: BMJ Open. 2025 Mar 6;15(3):e088921. doi: 10.1136/bmjopen-2024-088921 (PMC11887307; doi:10.1136/bmjopen-2024-088921)
Supplement: Supplementary file 4 [file bmjopen-2024-088921.reviewer_comments.pdf]

PEER REVIEW HISTORY

BMJ Open publishes all reviews undertaken for accepted manuscripts. Reviewers are asked to complete a checklist review form and are provided with free text boxes to elaborate on their assessment. These free text comments are reproduced below.

ARTICLE DETAILS

Title (Provisional)

Addressing the challenges and constraints of social protection policies for Peruvian women domestic workers: The ANITA project study protocol

Authors

Tenorio-Mucha, Janeth; García, Sebastián; Portocarrero, Jill; Lazo-Porras, Maria; Romero Rivero, Karina; Vera Tudela, David; Cárdenas, María Kathia; Pérez-León, Silvana; Aya Pastrana, Nathaly; Cuba-Fuentes, Maria Sofia; Hapsari, Ayu Pinky; Meaney, Christopher; Pinto, Andrew David; Cruzado, Viviana; Gupta, Archna

---

VERSION 1 - REVIEW

**Reviewer**	**1**
**Name**	**Thakkar, Shriya**
**Affiliation**	**Louisiana State University**
**Date**	**01-Aug-2024**
**COI**	**N/A**

The manuscript is not of sufficient quality and it needs a lot of work to reach a stage where it can be accepted for publication. I request the authors to carefully address the comments. Currently, it lacks clarity and feels rushed. Please take sufficient time and work further upon the manuscript.

1. The authors in line 21 introduce 'live-in' domestic workers all of a sudden. For clarity, the authors should distinguish early on what is the difference b/w live-in and live-out. From the introduction, it is unclear as to where the study context is. Although authors mention Peru, but they do not specify if that is the where the study context is. Given the topic of DWs which is highly subjective and context specific, it is important to foreground the context at the very outset. Further there are no mention of literature/existing work that has been done in the area (which is a lot of work) and how this study extends the current body of work.
2. The introduction is short and almost feels rushed. There is also lack of consistency—for instance, the authors mention the funding body without building any background. The authors mention that their study emerged from the need to improve working conditions of DWs, formalization etc., but multiple studies have already done this. So, the question then emerges, what is new in your research? I could not find the answer to this question from the introduction.

3. The introduction still needs a lot of redevelopments in terms of context, motivation, existing work—all of which will tie to the need for the readers to read beyond the introduction. Currently, this motivation was clearly lacking.
4. Same applies for the aims and objectives too which seemed a bit broad and addressing the questions which already has a lot of existing literature. One way to tackle this could be to present a good build-up about Peru. Is there anything typical/specific to Peru due to which this site was chosen? The authors should try to answer these questions when framing the research objectives.
5. Specify what sort of quantitative data you would be collecting—cross-sectional, longitudinal, surveys, or something else?
6. The authors mention that the qualitative findings “will explain” quantitative findings by exploring key informant’s experiences…I am curious to know how that will be achieved. Will you be asking similar questions during both data collection? And if so, your study would be prone to biases. What are the steps that you will take to minimize biases? The qualitative data collection should be free-flowing and not driven by the results of the quantitative data. And, if there were specific themes that you want to explore, it should be clearly specified. Currently, the process of data collection has serious problems and needs to be given another thought to tackle the questions posed above.
7. I like the demographic information provided for the face-to-face interviews which is clear and specific.
8. In 4.2, the authors mention their data sources, but this should also be underpinned above. Further, mention the type of both datasets, what all waves are being considered in the years mentioned—is it yearly?
9. Reading further on, some of the above questions are partially answered, however, as a reader, the problem remains of clarity. Whenever quantitative analysis is mentioned, it is important to state whether it is from a publicly sourced dataset etc. The reader should have some background at the very outset.
10. What kind of regressions will you be conducting? What will be your sample size for the quant data? How will you be handling missing data? Since you mentioned regression, you should explicitly state what will be your outcome variable, exposure variables, other important predictors, any moderators or mediators. Currently, all of the above information is missing.
11. There could be a subheading of exclusion criteria in the study.
12. 448 seems like a large sample size to conduct face-to-face interviews. What will be your estimated study time given the large N?
13. For phase 3, given the diverse group of participants, I am not sure if interviewing different individuals should be correct. How about focus group discussions which could be more meaningful.
14. For the data analysis of phase 3, are you hinting at intercoder reliability analysis? If yes, it was unclear and wasn’t mentioned.
15. Strengths and limitations were not expanded. The pointers should be further expanded.

**Reviewer**	**2**
**Name**	**Garabiles, Melissa**
**Affiliation**	**De La Salle University, Department of Psychology**

**Date**	**19-Aug-2024**
**COI**	**None**

The protocol is well-written, and the topic is important to study. I think the paper can be improved by adding more details about the context and the method.

Strengths and limitations: Please state them in complete sentences

Please provide more background about Peru and DWs. Apart from the Domestic Workers Law, are there other laws in Peru that aim to protect DWs or workers in general? Is Peru a signatory to the ILO Convention 189 or other DW-related international instruments?

Are there statistics on the demographic information of DWs? For example, what is the percentage of households that employ DWs? Are they internal or international migrants? How many are staying in their employers' homes vs staying outside? Among the latter, where do they typically stay? How many have children? What is their marital status? How many have written contracts? Etc.

How many DW organizations are there? How do they differ from each other? What percentage of DWs are part of an organization? Are the members and leaders all DWs themselves? If not, what is their occupation and how were they chosen to be leaders of a DW organization? Later in the protocol, it is mentioned that male DWs may be part of the study. Is there a difference in terms of work load/scope/conditions between male and female DWs in Peru?

Differentiate the co-researcher and advisory committee more. Add details on how the DWs were chosen.

Add details regarding the steering committee, such as what the key departments are and their mandate vis a vis DWs.

Add details regarding the exact items from the ENAHO and ENDES that will be used in the study, either in the text or in the appendix.

There is a lack of information about the measures. Expound on the measures that will be included in the survey, e.g., name of measure, authors/developers, scaling, scoring procedure, sample items, psychometric properties, etc.

Survey: Expound on the data analysis procedure

How would the co-researcher and advisory committees provide expert judgment regarding the survey? Provide details.

Would the DW participants in the qualitative interview be participants in the surveys as well?

Will the survey data inform the interview questions?

Will the co-researcher and advisory committee members critique the interview guides?

Will the surveys and interviews be conducted in-person, online, or both?

Provide details on the pre-established set of codes. Where did this come from? Will this be discussed with the committee members?

I suggest using a checklist like SRQR or COREQ to help ensure that a standard for reporting qualitative research is met.

For the second deliberative dialogue, having 18-26 participants seems to be a lot. With this number, how would the team ensure that each participant would be given the time and space to share their views? Further, the group will consist of people with varied occupational backgrounds. What will the team do so that certain occupations will not overpower or intimidate (e.g., policymakers) the others?

Expound on what the team did to make sure the informed consent forms were culturally sensitive and accessible.

In what language would each phase be?

Provide a section on the researchers' reflexivity, e.g., who has conducted DW research, if researchers are employer of DWs, connections with DW community, etc.

Discussion: It might be better to move parts that contextualize DWs in Peru to the Introduction

The tables/figures are nice and help clarify the method. Please indicate in the appropriate text that there are tables/figures that the reader can refer to.

VERSION 1 - AUTHOR RESPONSE

Reviewers' Comments:

1. The authors in line 21 introduce 'live-in' domestic workers all of a sudden. For clarity, the authors should distinguish early on what is the difference b/w live-in and live-out. From the introduction, it is unclear as to where the study context is. Although authors mention Peru, but they do not specify if that is the where the study context is. Given the topic of DWs which is highly subjective and context specific, it is important to foreground the context at the very outset. Further there are no mention of literature/existing work that has been done in the area (which is a lot of work) and how this study extends the current body of work.

   R. Thank you for the feedback. We have revised the introduction to include more detailed information on the country's context relevant to the study. Specifically, we have provided background on the social protection policies for domestic workers in Latin America and in Peru. We also included key statistics about working conditions after and during the pandemic. Additionally, we have incorporated key studies from the existing literature to better frame the ANITA project. This has allowed us to highlight gaps in the literature and underscore the importance of our research within the country's specific socio-economic and health framework. We believe these changes significantly improve the clarity and relevance of the introduction.

2. The introduction is short and almost feels rushed. There is also lack of consistency—for instance, the authors mention the funding body without building any background. The authors mention that their study emerged from the need to improve working conditions of DWs, formalization etc., but multiple studies have already done this. So, the question then emerges, what is new in your research? I could not find the answer to this question from the introduction.

   R. Thank you for the comment. We have now made the novelty of our research more explicit in the revised manuscript. Specifically, we highlight that our study uses a mixed-methods, a participatory action research approach, the respondent-driven sampling technic to recruit participants, and the knowledge translation activities. It can be read in paragraph 7 of the Introduction section.

3. The introduction still needs a lot of redevelopments in terms of context, motivation, existing work—all of which will tie to the need for the readers to read beyond the introduction. Currently, this motivation was clearly lacking.

   R. We understand the importance of a well-developed introduction and appreciate your feedback. As mentioned in our responses to previous comments, we have already addressed these concerns by improving the background, adding more detailed information on the country's specific context, and incorporating relevant literature to support the framing of the study. Furthermore, we have strengthened the motivation for our research by highlighting the novelty and gaps in the existing work, as well as explaining the need for this study.

4. Same applies for the aims and objectives too which seemed a bit broad and addressing the questions which already has a lot of existing literature. One way to tackle this could be to present a good build-up about Peru. Is there anything typical/specific to Peru due to which this site was chosen? The authors should try to answer these questions when framing the research objectives.

   R. We have revised both the introduction and the framing of the objectives. By refining the introduction, we believe the aims and objectives are now more focused and clearly distinguish our work from existing literature, addressing key questions relevant to the Peruvian context.

5. Specify what sort of quantitative data you would be collecting—cross-sectional, longitudinal, surveys, or something else?

   R. Thank you for the feedback. We are specifying the type of quantitative data to be collected. For phase 1, we will use annual survey databases from 2018 to 2022, and for phase 2, we will use a cross-sectional survey of women domestic workers.

6. The authors mention that the qualitative findings "will explain" quantitative findings by exploring key informant's experiences…I am curious to know how that will be achieved. Will you be asking similar questions during both data collection? And if so, your study would be prone to biases. What are the steps that you will take to minimize biases? The qualitative data collection should be free-flowing and not driven by the results of the quantitative data. And, if there were specific themes that you want to explore, it should be clearly specified. Currently, the process of data collection has serious problems and needs to be given another thought to tackle the questions posed above.

   R. Thank you for the comment. We better framed the qualitative methods. We have explained that the qualitative data will deepen the quantitative findings by exploring in detail the experiences and attitudes of key informants. In Section 4.4 Phase 3, we have detailed the themes to be explored, the design process of the interview guides and the process of data collection.

   The interview guides are structured in key themes, including access to social protection policies, perceptions of COVID-19 and its impact on the work of DWs, and views on Peruvian legislation for domestic work. These themes are intended to further explore notable findings from Phase 1 and 2 by exploring in detail experiences and attitudes of the participants. To ensure all interview

questions are both sensitive and culturally appropriate, the guides will be shared with members of our Advisory and Steering Committees for review before data collection.

7. I like the demographic information provided for the face-to-face interviews which is clear and specific.

   R. We appreciate the feedback.

8. In 4.2, the authors mention their data sources, but this should also be underpinned above. Further, mention the type of both datasets, what all waves are being considered in the years mentioned—is it yearly?

   R. Thank you for the comment. We have provided details about both datasets, which are annual surveys collected by the National Institute of Statistics. The databases are publicly available and we will use information from 2018 to 2022.

9. Reading further on, some of the above questions are partially answered, however, as a reader, the problem remains of clarity. Whenever quantitative analysis is mentioned, it is important to state whether it is from a publicly sourced dataset etc. The reader should have some background at the very outset.

   R. Thank you for pointing this out. We have clarified the source of the data used in our quantitative analyses in the revised introduction and methodology sections. By providing this background early on, we aim to improve the overall clarity and transparency of our research approach, allowing readers to better understand the foundation of our analysis.

10. What kind of regressions will you be conducting? What will be your sample size for the quant data? How will you be handling missing data? Since you mentioned regression, you should explicitly state what will be your outcome variable, exposure variables, other important predictors, any moderators or mediators. Currently, all of the above information is missing.

    R. We've included a detailed information for the plan analysis. The total sample is of 4216 participants who self-report working as DWs. We will use logit regressions with four main outcomes: weekly hours worked, hourly wage, the presence of illness symptoms, and healthcare access. Control variables will include sociodemographic factors such as place of residence, ethnic/racial self-identification, education, age, poverty status, migrant status, health insurance coverage, number of children, and marital status. Additionally, we will consider three year-specific dummy variables (for 2020, 2021, and 2022) to determine temporal moderation.

11. There could be a subheading of exclusion criteria in the study.

    R. Thank you for your comment. We haven't added a subheading as such, but we have added some text that mentions the exclusion criteria. We will exclude women who do unpaid domestic work or who do not have this activity as their main income.

12. 448 seems like a large sample size to conduct face-to-face interviews. What will be your estimated study time given the large N?

    R. Thank you for pointing this out. The sample size takes into account the effect of the respondent-driven sampling approach. We expect that the referral system and the dual incentive system will increase the chances of recruitment and reduce the time needed for data collection. We now include the planned timeline for each phase in the 4.1. Research Design section. For Phase 2, we have planned 10 months, 4 of which will be dedicated to data collection only.

13. For phase 3, given the diverse group of participants, I am not sure if interviewing different individuals should be correct. How about focus group discussions which could be more meaningful.

    R. We appreciate your comment, but we consider that individual interviews will allow us to gather personal beliefs and experiences in privacy. The decision to conduct in-depth interviews rather than using other data collection methods is grounded in two key considerations. First, interviews allow us to capture the personal stories of DW and leaders of DW organizations, which is essential to understanding the unique experiences and challenges they face in both their daily lives and leadership roles. Conducting a focus group would not enable the same depth and authenticity in these individual narratives, as the group dynamic could limit the expression of nuanced personal and private experiences. Second, we opted for interviews with employers and civil servants due to the limited availability of time these participants typically have. From a logistical perspective, individual interviews were the most viable and effective option, enabling us to access their perspectives without compromising the quality or depth of the collected data.

14. For the data analysis of phase 3, are you hinting at intercoder reliability analysis? If yes, it was unclear and wasn't mentioned.

    R. We will not conduct an intercoder reliability analysis. In our data analysis, we clarified that the initial coding framework will be based on Moss and SDOH frameworks, which guide our study. Additional codes will then be added through an iterative review process.

15. Strengths and limitations were not expanded. The pointers should be further expanded.

    R. Thank you for your comment. We have improved the Strengths and Limitations section. They are placed after the Abstract, according the BMJ journal guidelines.

16. Please provide more background about Peru and DWs. Apart from the Domestic Workers Law, are there other laws in Peru that aim to protect DWs or workers in general? Is Peru a signatory to the ILO Convention 189 or other DW-related international instruments?

    R. As noted in our previous responses, we have addressed these concerns by enhancing the introduction, providing details about national legal framework from domestic workers.

17. Are there statistics on the demographic information of DWs? For example, what is the percentage of households that employ DWs? Are they internal or international migrants? How many are staying in their employers' homes vs staying outside? Among the latter, where do they typically stay? How many have children? What is their marital status? How many have written contracts? Etc.

    R. Thank you for these valuable questions. In response, we have added relevant demographic information to the Introduction section. Our project acknowledges the gaps in the existing literature, which is precisely why we aim to collect specific data through both the survey and qualitative interviews. This approach will allow us to address most of the questions raised by the reviewer and provide a more comprehensive understanding.

18. How many DW organizations are there? How do they differ from each other? What percentage of DWs are part of an organization? Are the members and leaders all DWs themselves? If not, what is their occupation and how were they chosen to be leaders of a DW organization? Later in the protocol, it is mentioned that male DWs may be part of the study. Is there a difference in terms of work load/scope/conditions between male and female DWs in Peru?

    R. Thank you for the comment. We provided details about DW organizations in the Participatory action research section. There are 9 organizations registered in the Ministry of Labor, there are two

types: unions and training and advocacy organizations. The members and the leaders are current or former DWs.
There are indeed male DWs, they make up about 4% of the workforce. We recognize the importance of their role, but we will not include them in the study. This project was developed with a gender-responsive approach, taking into account the predominant role of women in domestic work and the invisibility of the work itself.

19. Differentiate the co-researcher and advisory committee more. Add details on how the DWs were chosen.

    R. To better differentiate the committees we include Figure 1 which summarizes the characteristics of the members and key roles of the Steering Committee, Advisory Committee, and Co-Researchers Committee.

20. Add details regarding the steering committee, such as what the key departments are and their mandate vis a vis DWs.
    R. Thank you for the comment. We have specified the departments and institutions that are considered part of the Steering Committee. They include the Department of Fundamental Rights of the Ministry of Labor, the National Office of the Pension System, the National Superintendence of Labor Inspection, and the Social Security.

21. Add details regarding the exact items from the ENAHO and ENDES that will be used in the study, either in the text or in the appendix.

    R. Thank you for the comment. We have included a table with the variables and items from the ENAHO and ENDES in the Supplementary Material 1.

22. There is a lack of information about the measures. Expound on the measures that will be included in the survey, e.g., name of measure, authors/developers, scaling, scoring procedure, sample items, psychometric properties, etc.

    R. Thank you for the comment. We have detailed information about the measures, questions, and questionnaires properties in the Supplementary Material 2.

23. Survey: Expound on the data analysis procedure

    R. Thank you for the comment. We better explained the analysis procedure which will consider the respondent-driven sampling design, using and RDS-II adjustment. It takes into account a weight to each participant based on their sampling probability according their network size, that is DWs who are more connected to other DWs will received a lower weight than those who are less connected. We will present simple estimates of counts/percentages for categorical variables, and means/standard deviation or median/interquartile range for continuous variables depending on the distribution.

24. How would the co-researcher and advisory committees provide expert judgment regarding the survey? Provide details.

    R. Thank you for pointing this out. We now have described the procedure in detail. We will organize meetings with the co-researchers committee to discuss the appropriateness of the questionnaires and also, we will conduct a pilot with the advisory committee to refine the length and language of the questions.

25. Would the DW participants in the qualitative interview be participants in the surveys as well?

R. To identify potential participants, we will use stakeholder mapping and feedback from the three committees. We will recruit participants by snowballing. Some DWs interviewed may have also participated in Phase 2; however, we will not actively seek them out, as their identities will remain confidential.

26. Will the survey data inform the interview questions?

    R. The interview guides are structured in key themes, including access to social protection policies, perceptions of COVID-19 and its impact on the work of DWs, and views on Peruvian legislation for domestic work. These themes are intended to further explore notable findings from Phase 1 (secondary data analysis) and Phase 2 (survey) by exploring in detail experiences and attitudes of the participants. We have detailed this information in the revised version.

27. Will the co-researcher and advisory committee members critique the interview guides?

    R. To ensure all interview questions are both sensitive and culturally appropriate, the guides will be shared with members of our Advisory and Steering Committees for review before data collection. This iterative process allows committee members to provide feedback, which the research team will then analyze thoroughly. Based on this input, the final version of the guides will be carefully edited to ensure they elicit meaningful, respectful, and insightful responses from participants. We have detailed this information in the revised version.

28. Will the surveys and interviews be conducted in-person, online, or both?

    R. The Phase 2 survey will be conducted in-person, while the Phase 3 interviews will be conducted both in-person and online, depending on the logistical and contextual needs of the participants and the study. We have detailed this information in the revised version.

29. Provide details on the pre-established set of codes. Where did this come from? Will this be discussed with the committee members?

    R. W clarified that the initial coding framework will be based on Moss and SDOH frameworks, which guide our study. Additional codes will then be added through an iterative review process. We did not contemplate to discuss the emerging codes with the committee members.

30. I suggest using a checklist like SRQR or COREQ to help ensure that a standard for reporting qualitative research is met.

    R. Thank you for the suggestion. While we do not use the COREQ checklist directly, we have incorporated key elements it recommends, including details on the research team and reflexivity, theoretical framework, participant selection, interview guide, and coding derivation.

31. For the second deliberative dialogue, having 18-26 participants seems to be a lot. With this number, how would the team ensure that each participant would be given the time and space to share their views? Further, the group will consist of people with varied occupational backgrounds. What will the team do so that certain occupations will not overpower or intimidate (e.g., policymakers) the others?

    R. Thank you for raising these important points. We recognize that a group of 18–26 participants requires careful facilitation to ensure everyone has the opportunity to contribute. To address this, we will employ a structured dialogue format that includes small-group discussions to allow each participant to share their perspectives in a more intimate setting before bringing key insights to the larger group. Each small group will have a trained facilitator to guide the discussion and ensure balanced participation.

Additionally, to minimize any potential power imbalances among, facilitators will be briefed to actively encourage contributions from all attendees, paying particular attention to the voices of individuals who may feel less empowered, such as domestic workers. By setting ground rules for respectful and inclusive dialogue at the beginning of the session, we aim to create a safe environment where all participants feel valued and respected, regardless of their occupational role.

32. Expound on what the team did to make sure the informed consent forms were culturally sensitive and accessible.

    R. Thank you for the comment. We have detailed characteristics and procedures to obtained the informed consent We explained the special considerations for individuals with reading difficulties and for adolescents.

33. In what language would each phase be?

    R. The study will be conducted in Spanish. We have added this information.

34. Provide a section on the researchers' reflexivity, e.g., who has conducted DW research, if researchers are employer of DWs, connections with DW community, etc.

    R. We appreciated the suggestion. We have added a subheading of Reflexivity in the end of the Ethic and Dissemination section, describing of the expertise of the team members who have designed the project. We would appreciate it if the editor would indicate that we should move this subtopic to another section of the manuscript.

35. Discussion: It might be better to move parts that contextualize DWs in Peru to the Introduction

    R. We have removed Discussion section.

36. The tables/figures are nice and help clarify the method. Please indicate in the appropriate text that there are tables/figures that the reader can refer to.

    R. We have verified that all the tables and figures are referenced in the text.

VERSION 2 - REVIEW

**Reviewer**	**1**
**Name**	**Thakkar, Shriya**
**Affiliation**	**Louisiana State University**
**Date**	**03-Dec-2024**
**COI**	

Overall, the manuscript would benefit from careful rewriting and editing to address minor grammatical and punctuation errors throughout the text. Additionally, for variables such as hours worked and hourly wage, which appear to be continuous, please clarify how logistic regression is applicable and specify the threshold used for categorization. Providing more

detailed information about the data sources—such as when the data collection began, the years covered, and any relevant context—would enhance the clarity and transparency of the study. Furthermore, it would be helpful to explain how you plan to ensure that the qualitative analysis includes the same individuals as those in the quantitative dataset. Alternatively, you may wish to consider treating the qualitative and quantitative components as two separate studies. This is an important aspect to reflect on as you refine the manuscript.

VERSION 2 - AUTHOR RESPONSE

Reviewer: 1

Overall, the manuscript would benefit from careful rewriting and editing to address minor grammatical and punctuation errors throughout the text. Additionally, for variables such as hours worked and hourly wage, which appear to be continuous, please clarify how logistic regression is applicable and specify the threshold used for categorization. Providing more detailed information about the data sources—such as when the data collection began, the years covered, and any relevant context—would enhance the clarity and transparency of the study. Furthermore, it would be helpful to explain how you plan to ensure that the qualitative analysis includes the same individuals as those in the quantitative dataset. Alternatively, you may wish to consider treating the qualitative and quantitative components as two separate studies. This is an important aspect to reflect on as you refine the manuscript.

Response:

Thank you for your feedback on our manuscript. We have carefully considered each of the points raised and made appropriate revisions to address them. Below, we provide a detailed response:

1. *Grammar and editing*

We carefully reviewed the manuscript and made significant revisions to address grammatical and punctuation errors throughout the text. We have also improved clarity and readability to ensure a polished final document.

2. *Threshold for continuous variables*

The thresholds are now explicitly mentioned in the revised manuscript. They are based on what is stated in the Peruvian Domestic Workers Law.

3. *Details about the data*

We added detailed information about the data sources, including when data collection began, the time period covered, and relevant contextual information.

4. *Relationship between qualitative and quantitative participants*

In the Sample size and Recruitment sub-heading of Phase 3, we mention that the qualitative participants are not necessarily the same as those included in the quantitative dataset. While we do not exclude the possibility of overlap, we will not actively seek to include the same individuals. This approach ensures flexibility in participant recruitment.

5. *Integration of quantitative and qualitative components*

We respectfully disagree with the suggestion to treat the quantitative and qualitative components as entirely separate studies. Instead, we have maintained an integrated mixed-methods approach. We consider that combining both approaches allows us to leverage the strengths of each, providing a more

comprehensive understanding and facilitating richer interpretations of the findings. The mixed-method approach ensures alignment with the study’s overarching objectives, avoiding fragmentation of the research.
